# Importance of dashboard camera (Dash Cam) analysis in fatal vehicle–pedestrian crash reconstruction

**DOI:** 10.1007/s12024-021-00382-0

**Published:** 2021-05-19

**Authors:** Elena Giovannini, Arianna Giorgetti, Guido Pelletti, Alessio Giusti, Marco Garagnani, Jennifer Paola Pascali, Susi Pelotti, Paolo Fais

**Affiliations:** 1 Department of Medical and Surgical Sciences, Unit of Legal Medicine, University of Bologna, Via Irnerio 49, 40126 Bologna, Italy; 2 Department of Cardiologic, Thoracic and Vascular Sciences, University of Padova, Via Giustiniani, 2 35127, Padova, Italy

**Keywords:** Forensic pathology, Traffic crash, Dash Cam, Privacy right

## Abstract

The reconstruction of dynamic of traffic injuries remains a challenge in forensic pathology and is often based on circumstantial data. Dash Cams are digital video recorders which can be located inside a vehicle and continuously record the view through the windscreen, thus providing objective evidence. Here we present the case of a traffic crash in which a pedestrian was hit by an articulated lorry. The analysis of a video recorded from a Dash Cam retrieved inside the vehicle during the death scene investigation (DSI) was crucial in the reconstruction of the manner of death. Indeed, the death, which was initially assumed to be accidental, was finally deemed as a suicide on the basis of the video recording, which showed an intentional and sudden rush of the victim to the middle of the roadway. Advantages and disadvantages of the use of Dash Cams will be discussed, focusing on the profound differences in the related national and international regulations. Based on the present case, in traffic crashes, the search for Dash Cams during the DSI may be recommended and the video recordings should be analyzed in the setting of a multidisciplinary and multimodal evaluation of the case, for a proper reconstruction of the facts.

**Supplementary Information:**

The online version contains supplementary material available at 10.1007/s12024-021-00382-0.

## Introduction

The reconstruction of the dynamics of a traffic crash is usually achieved by interviewing involved subjects, eyewitnesses and through post-collision mechanical or engineering examination. For some incidents, circumstantial data may be enough to resolve questions concerning the crash **[**1]. However, sometimes circumstantial data is missing, there are no eyewitnesses, or drivers and passengers do not survive to tell their stories or they have difficulty recalling the details of the events as a result of the injuries sustained in the collision. In addition, especially when the collection of statements from eyewitnesses is delayed, the recollection of the events becomes increasingly difficult over time [2–5]. Sophisticated computer software programs and reconstruction models can also be applied, with good estimation of the dynamics of the crash, though the degree of certainty required for criminal proceedings is rarely achieved [6, 7]. Other sources for pre-crash information (e.g. vehicle speed, acceleration, brake application etc.) could be obtained from event data recorders (EDRs). Even the airbag opening might trigger the collection of such data, which could be used in the incident investigation [8, 9].

Dashboard-mounted cameras or “Dash Cams” are a digital video recorder (DVR) which can be installed on the dashboard or on the windscreen, by means of a suction cup, and continuously record the view through the glass of a windscreen [2, 10]. Since the beginning of their use in the 1980s in Texas, such video recordings have increased the safety of officers working in remote areas [11]. As the technology became cheaper, Dash Cams also became accessible to other drivers and the number of vehicles mounting Dash Cams rapidly increased [12]. Some modern technologies referred to as “Dual Dash Cams”, include a second camera to record the interior and/or rear of the car. Furthermore, the more sophisticated Dash Cams allow the recording of other specific data such as GPS data files and measurements of acceleration and deceleration (g-force), speed and steering angle of the vehicle [8–10] and this could make the reconstruction of the crash events easier [13]. However, Dash Cams might attract negative attitudes due to privacy concerns, thus in many countries they are legal only under determined conditions, or even illegal.

The aim of this study is to present a case in which the video-recording captured from a Dash Cam was crucial to properly reconstruct the events of a crash. In order to verify the inevitability of the collision and the reliability of Dash Cam data, the assessment of the dynamics was performed by comparing Dash Cam footage with standard mathematical formulas. The benefits and the legal issues related to the use of Dash Cams will be discussed.

## Case report

### History and death scene investigation

**Fig. 1:**
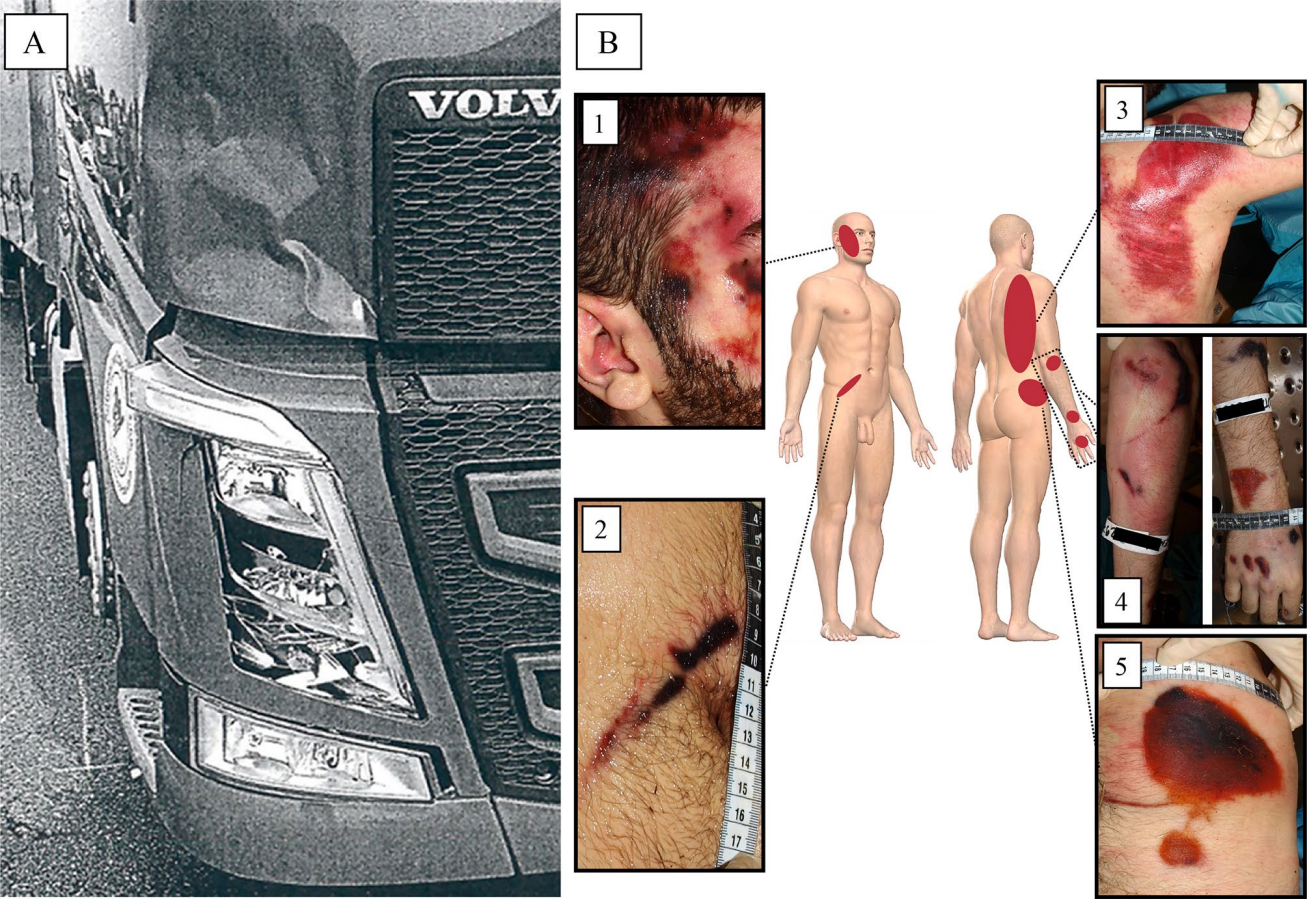
**A:** Damage on the anterior right side of the lorry. **B**: Injuries found on the body of the pedestrian at post-mortem examination. **B1** right side of the head; **B2** right umbilical region; **B3** posterior surface of the right shoulder; **B4** right elbow, forearm and hand; **B5** right side of the pelvis

As reported by an eyewitness, a 30-year-old male driver ran off a straight road, got out of his severely damaged car and walked for a while on the roadside. The man was in an apparent state of confusion. After a few minutes walking, he was hit by an articulated lorry (Volvo FH 460) that was driving in the opposite direction. At the death scene investigation (DSI), the body of the victim was found in the field on the side of the road, about 10 m from the point of presumed impact. The anterior right headlamp and the anterior right part of the bumper of the truck were damaged after the crash (Fig. 1A). Law enforcement officers retrieved a Dash Cam from the dashboard of the articulated lorry and cannabis products inside the car of the victim. According to circumstantial data, the man did not suffer from depression or other psychiatric diseases.

### Autopsy

**Fig. 2:**
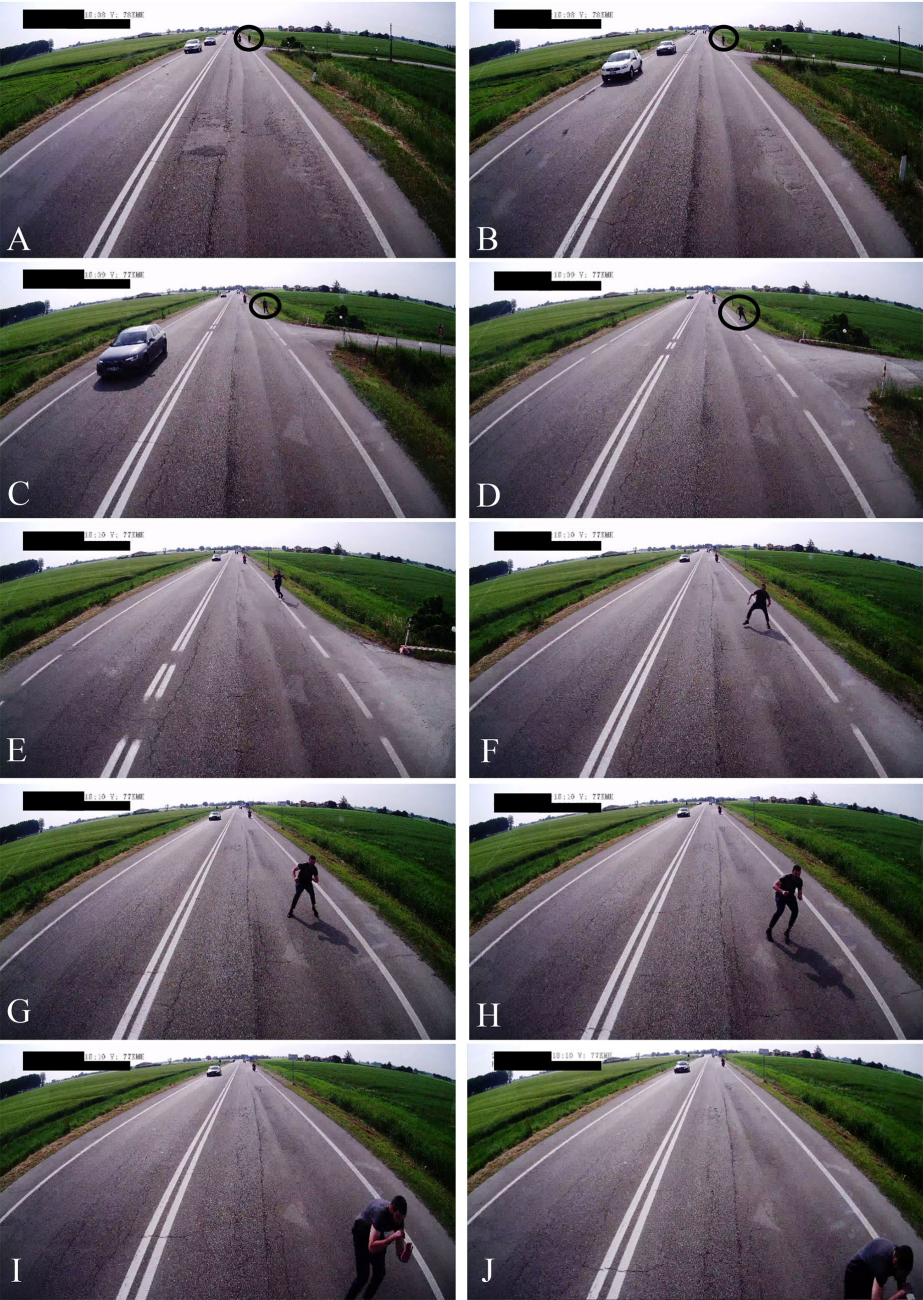
Picture sequence taken from the Dash Cam of the articulated lorry during the victim's investment (**a**-**j**). The time (minutes:seconds) and the speed of the vehicle (Km/h) are shown in each frame in the upper-left side

**Fig. 3:**
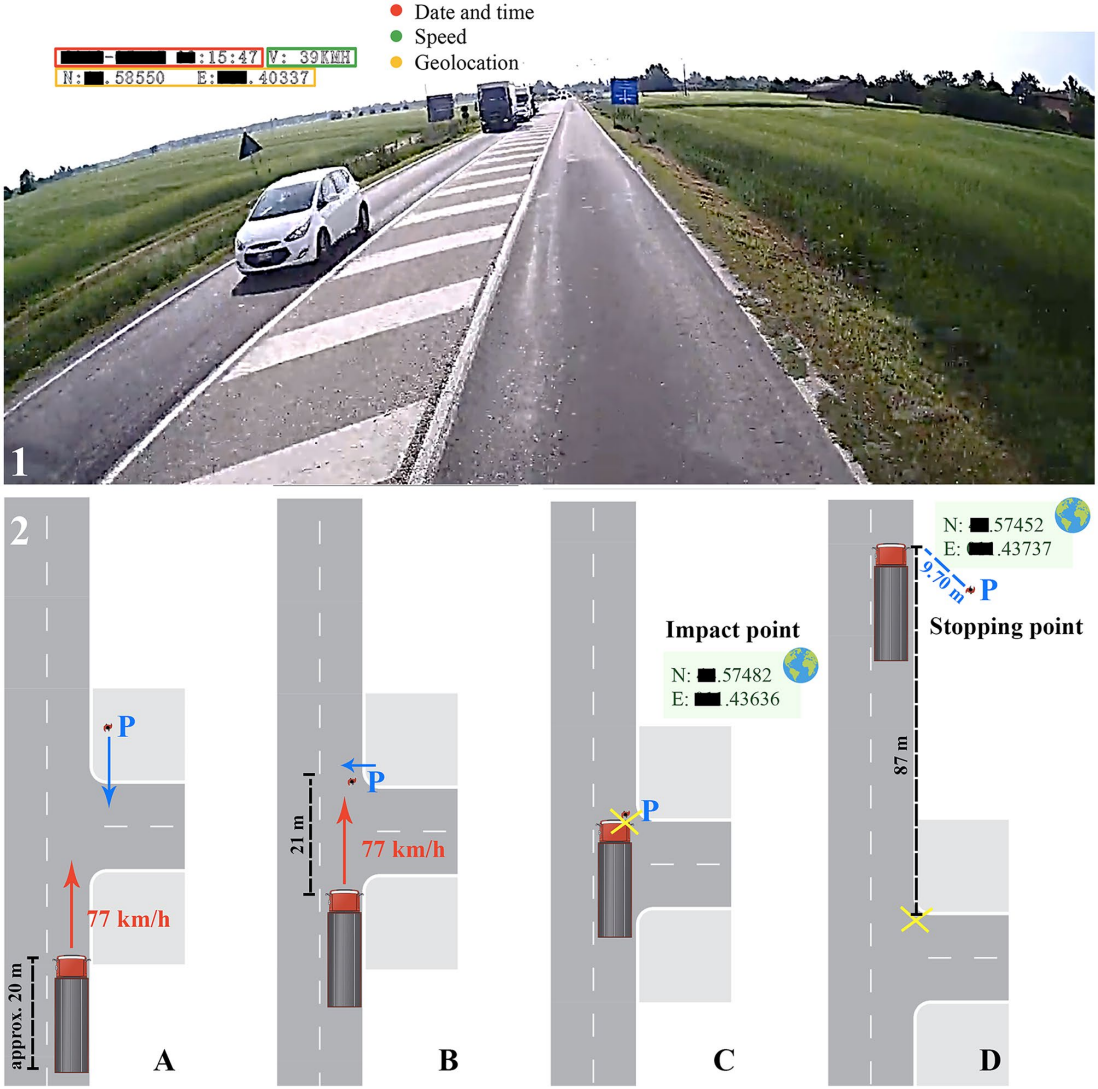
1: A frame of the video recorded by the Dash Cam. **2** (A-D): Reconstruction of the dynamics. E: east; N: north; P: pedestrian

**Fig. 4:**
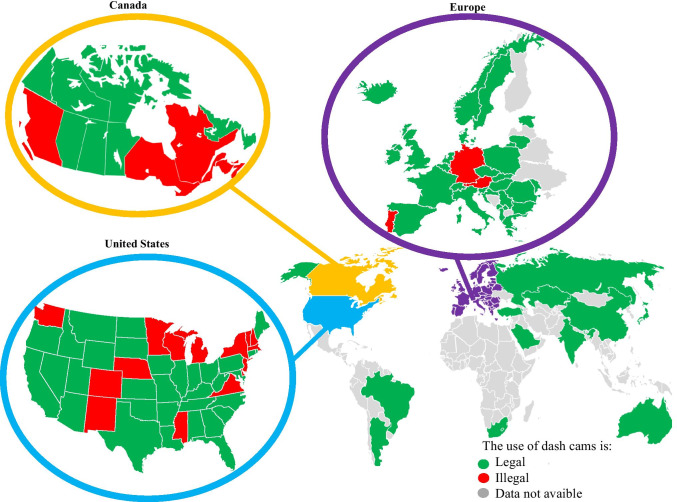
Legal regulation on the use of Dash Cams across the World

Medico-legal autopsy was performed 24 h post-mortem. The external examination demonstrated multiple extensive traumatic injuries. Abrasions and bruises were observed on the right side of the head (Fig. 1B, 1), on the right umbilical region (Fig. 1B, 2), on the posterior surface of the right shoulder (Fig. 1B, 3), on the right elbow, forearm and hand (Fig. 1B, 4) and on the right lateral side of the pelvis (Fig. 1B, 5). The cadaveric section showed a subarachnoid extensive hemorrhage at the right parieto-temporo-occipital side, a displaced temporo-parietal right fracture and a displaced fracture of the base of the skull with multiple bone fragments. The brain showed multiple contusions, mainly involving the temporal and parietal areas. Lung contusions and liver lacerations were additionally observed. The cause of death was deemed as polytrauma with neurogenic shock.

### Toxicological analyses

Toxicological analysis was performed on post-mortem blood and on urine. A general enzyme immunoassay screening and a specific research for cannabinoids and alcohol by GC–MS [14] tested positive for 11-Nor-9-carboxy-Δ9-tetrahydrocannabinol (THC-COOH) in urine and central blood (112 ng/mL). A quali-quantitative screening for psychoactive drugs and for 150 novel psychoactive substances (NPS, listed in Supplementary material 1) was additionally performed on blood samples by means of ultra-performance liquid chromatography tandem mass spectrometry (UPLC-MS/MS) with fully validated methods according to International Guidelines [15–17]. Samples were found negative for all tested compounds.

### Dash Cam recording analysis

The images captured by the Dash Cam provided information about the date, time (in hours, minutes and seconds), speed and geolocation (using north and east coordinates) of the vehicle (Figs. 2 and 3.1). The truck was travelling at a speed of 77 km/h on a major road with a speed limit of 70 km/h (Fig. 3.2A ). The road lacked sidewalks and was very narrow. After walking for a while on the right roadside, the man suddenly rushed at the middle of the roadway (Fig. 3.2B) and remained there. He turned the trunk and the head a little to the left and squatted, raised the right forearm to the head level with the exposure of the dorsal side of the right hand, waiting for the impact, with no attempt to get out of the way of the truck (Fig. 2A-J). At this moment, through geolocation coordinates analysis, it was possible to calculate that the articulated truck was located at a distance of about 21 m from the man in the middle of the road. The collision between the body of the man and the front of the articulated truck occurred in a timeframe equal to or less than 1 s (decimals are not available) (Fig. 3.2C). The speed of the articulated truck began decreasing approximately one second after the impact and the vehicle stopped 7 s after the impact (Fig. 3.2D).

In addition, the Dash Cam analysis provided the geolocation of the point of impact with the pedestrian and the final stopping point of the articulated truck. By using the geolocation in satellite navigation systems (e.g. Google Maps), a stopping distance of 87 m was reconstructed between the impact point and the stopping point.

In general, stopping distance, which includes the time it takes to react to a hazard (reaction distance) and the time it takes for the brakes to stop the car (braking distance) [18–20], may be calculated by mathematical formulas.

$$Stopping\;distance=Reaction\;distance(RD)+Braking\;distance(BD)$$

The RD and BD for a vehicle driving along a level road can be roughly determined by applying the following formula:

$$RD\;\left(m\right)=v\cdot t$$

$$BD\;(m)=0.039\cdot\frac{V^2}a$$

where:

*t* = reaction time

*v* = speed (m/s)

*V* = speed (km/h)

*a* = deceleration (m/s^2^)

Thus, in this case, basing on the speed at the moment of impact (77 km/h or 21.39 m/s) and taking into consideration the average reaction time reported in the literature in a healthy subject (about one second) [20], it is possible to calculate the RD:

$$RD=v\cdot1\;s=21.39\frac ms\cdot1\;s=21.39\;m$$

To calculate BD it is necessary to know the deceleration of the articulated truck, which occurred in 7 s.

$$a=\frac{\Delta v}{\Delta t}=\frac{0\frac{km}h-\left(-77\frac{km}h\right)}{5\;s}=\frac{0\frac ms-\left(-21.39\frac ms\right)}{6\;s}=3.56\frac m{s^2}$$

$$BD=0.039\cdot\frac{\left(77\;km/h\right)^2}{3.56\;m/s^2}=61.7m$$

By adding RD and BD, the stopping distance was calculated as:

$$Stopping\;distance=RD+BD=21.39\;m+61.7\;m=83.09\;m$$

The same formula also allowed us to infer that the truck would have only been able to come to a stop and avoid the collision if it had been travelling at a speed lower than 8 km/h (i.e. with a stopping distance of 21 m), even if we allowed that braking occurred simultaneously with the sudden rush of the pedestrian into the middle of the road (i.e. an unlikely 0 s reaction time).

## Discussion

In the present case, law enforcement officers initially assumed that the death was accidental, because the lorry was travelling over the speed limit and occupying most of the roadway, the victim had no history of psychiatric illnesses, and had never shown suicidal intentions. However, the analysis of recordings acquired through the Dash Cam mounted on the truck’s dashboard showed an intentional and sudden rush of the victim to the middle of the roadway, with no attempt to avoid the collision. The reasons for this behavior are still a matter of debate. A comprehensive toxicological analysis was consistent with a previous exposure to cannabis. Although psychoactive drugs and NPS have been directly or indirectly associated with serious adverse neuropsychiatric effects, including inexplicable suicidal behaviors [21–23], neither recent consumption of any tested psychoactive drugs nor NPS were found.

Vehicle speed is well-known to influence the crash risk and to increase the unavoidability of a collision. The truck was travelling slightly above the speed limit, however this was irrelevant. Indeed, even at a lower speed (i.e. below the speed limit), given the sudden swerving of the subject, it would have been impossible to stop in time to avoid the collision.

Analysis of the DVR recording showed it took 2 s from the appearance of the pedestrian in the middle of the road to the first decrease of the truck’s speed (i.e. the application of brakes). Considering an average reaction time (to brake) of 1 s, it is likely that the driver only realized what happened after the collision; if he had reacted immediately, braking would have started around the time of impact. However, in the absence of braking sensors, this is only speculation. Vehicle factors, such as the weight of the truck, may also result in a delayed decrease in the truck’s speed despite timely braking.

As demonstrated by this case, DVR may be an important tool for clarifying the events of a crash, particularly in regard to impact dynamics and the manner of death, with important consequences for any resulting criminal charges.

Indeed, Dash Cams have been reported as being useful in investigating traffic crashes, in protection from fraud, investigations of parking collisions, in providing a record of road crimes, recording in-car driver activity, and in capturing the unexpected [12, 24, 25]. However, their use has raised several questions and discussions mainly related to the right to informational privacy or the inviolability of private life, established by international acts including the European Convention [26, 27]. Austria, Germany and Luxembourg have forbidden the use of Dash Cams, considering the right of informational self-determination (i.e. the right to choose which information to disclose to others) as prevailing on the advantages of Dash Cams, since the recording is movable and unannounced, so that filmed people cannot give a consent. In other countries, e.g. Lithuania, the United Kingdom and Belgium, the protection of the public interest prevails over the right to privacy and Dash Cam recordings are allowed, taking into account the proportionality principle, i.e. the use is permitted, when necessary, in a litigation to protect one’s rights and less intrusive means are unavailable [12].

In some countries the use of Dash Cams is encouraged by national government departments, insurance agencies and/or by business owners and drivers who want to supervise the actions of their employees. Nearly every country of the World has different laws regulating the installation of Dash Cams and the use of their recordings (Fig. 4), as detailed in Supplementary material 2. Given that this issue is still a matter of debate, in those countries in which Dash Cams are not explicitly banned, and when allowed by the Judicial Authority, videos and information recorded by Dash Cams should be included within the multidisciplinary and multimodal evaluation of traffic injury cases. The case presented in this paper underlines the importance of performing a comprehensive DSI in traffic crashes [28, 29], which should not only include the analysis of the exterior of the involved vehicles, to assess the sites of impact, but also the inside, to search for Dash Cam or built-in cameras.

Further controversy is raised by the possible artefacts and distortion of Dash Cam recordings, which can be classified into extrinsic or intrinsic, depending on whether the factor influencing the camera is external (e.g. weather conditions), or a component of the camera itself (e.g. the settings) that determine a perceptive distortion of the image [10, 30–34]. Since these factors might affect the reliability of data, when such technological devices are available in the course of the investigations, a forensic image analysis, i.e. the evaluation of image and recordings quality, artefacts and distortion [6, 30], is suggested and each piece of evidence should be weighed accordingly during the trial or criminal proceedings.

In the present case, it is interesting to note that the value of stopping distance as calculated by mathematical formulas, i.e. on the basis of time and speed, was in accordance with that derived from the geolocation coordinates. Slight differences between the two values might be due to the approximations used in the mathematical formula, which do not take into consideration additional factors (e. g. roadway grade and level road). The coherence between all these values can also be crucial in the forensic contest as evidence that the Dash Cam has not been manipulated and there are not any artifacts. While the distance between two points calculated through geolocation is precise and accurate, the identification of the specific impact point by geolocation services (Google maps/earth) can be affected by planimetric bias [35]. In fact, the video shows the exact point of impact at a road intersection, while geolocation identifies it 10 m behind that point.

The reconstruction of a traffic crash can be challenging [36]. In the setting of traffic crash followed by litigation or criminal proceedings, the analysis of recordings acquired through the Dash Cam may provide further evidence and help forensic experts in the interpretation of the dynamics of the events, taking into consideration that Dash Cam recordings are just one of the many circumstantial elements to be weighed in the trial.

## Conclusion

The indiscriminate use of Dash Cams and of the recorded videos pose serious risks of violation of privacy and privacy laws. On the other hand, as was seen in this case, Dash Cams can help forensic experts in the reconstruction of road crashes, preventing distortion of the facts which could lead to a possible miscarriage of justice. The recording of the Dash Cam in this case was crucial in suggesting an alternative manner of death and in providing helpful data to the judicial authority. Based on the present case, in traffic crashes, the search for Dash Cams during the DSI is recommended from the start of the investigations and, when allowed by the judicial authority, the video recordings should be analyzed in the setting of a multidisciplinary and multimodal evaluation of the case, for a proper reconstruction of the facts.

## Key points

1. Dash Cam recordings may be crucial in the reconstruction of traffic fatalities.
2. Issues regarding the right to privacy and the use of Dash Cams are a matter of debate.
3. The search for Dash Cams during the death scene investigation is strongly recommended.
4. Dash Cam video should be considered from the beginning of an investigation.

## Supplementary Information

Below is the link to the electronic supplementary material.

Supplementary file1 (DOCX 21 KB)

Supplementary file2 (DOCX 56 KB)
