# Supplementary material for: Importance of dashboard camera (Dash Cam) analysis in fatal vehicle–pedestrian crash reconstruction
Source: Forensic Sci Med Pathol. 2021 May 19;17(3):379–87. doi: 10.1007/s12024-021-00382-0 (PMC8413177; doi:10.1007/s12024-021-00382-0)
Supplement: Supplementary file 1 — Supplementary file1 (DOCX 21 KB) [file 12024_2021_382_MOESM1_ESM.docx]

**Supplementary material (1) to:**

**Importance of dashboard camera analysis in fatal vehicle-pedestrian crash reconstruction**

List of Novel Psychoactive Substances included in the analytical method (semi-systematic names, alphabetical order).

(±)-*cis*-3-methyl norfentanyl, (±)-*trans*-3-methyl norfentanyl, αET, β-Hydroxy fentanyl, β-hydroxythiofentanyl, β-phenyl fentanyl, 3,4-DMMC, 4-AcO-DiPT, 4-ANPP, 4-FMC (4-Fluoromethcathinone), 4-HTMPIPO, 4F-MDMB-BINACA, 4-MEC (4-Methylethcathinone), 5-APB/6-APB, 5-Cl-THJ 018, 5-EAPB, 5F-AB-001, 5F-AB-PICA (5F-ABICA), 5F-AB-PINACA, 5F-ADB, 5F-ADB-PICA (5F-ADBICA), 5F-ADB-PINACA, 5F-AKB-48 (5F-APINACA), 5F-AMB-PINACA, 5F-APP-PICA (PX-1), 5F-APP-PINACA (PX-2), 5F-CUMYL-PINACA, 5F-EMB-PINACA, 5F-JWH-412, 5F-MDMB-P7AICA, 5F-MDMB-PICA, 5F-MDMB-PINACA (5F-ADB) , 5F-NNEI 2'-Naphthyl Isomer, 5F-PCN (5F-MN-21), 5F-PY-PICA, 5-MAPB/6-MAPB, 5-MeO-AMT, 5-MeO-DALT, 5-MeO-DMT, 5-MeO-DPT, 5-MeO-MiPTAB-CHMINACA, A-796,260, A-834,735, AB-001, AB-005, AB-005-azepane, AB-BICA, AB-CHMICA, AB-FUBICA, AB-FUB7AICA (AB-7-FUBAICA), AB-FUBINACA, AB-FUBINACA 2/3-fluorobenzyl isomers, AB-PICA, AB-PINACA, Acetyl Fentanyl, Acetyl norfentanyl, ADB-BICA, ADB-BINACA, ADB-CHMICA, ADB-FUBICA, ADB-FUBINACA, ADB-PICA, ADB-PINACA, AKB-48 (APINACA), AKB-57 (APINAC), Alfentanyl, AM-1220 azepane, AM-1235, AM-1241, AM-1248, AM-1248 azepane, AM-2201, AM-2201 indazole carboxamide, AM-2232, AM-2233, AM-2233 azepane, AM-630, AM-679, AM-694, AMB-CHMICA, AMB-CHMINACA, AMB-FUBICA, AMB-FUBINACA, AMB-PICA, AMB-PINACA, APP-FUBINACA, BB-22, Buphedrone, Butylone, Butyryl fentanyl, Butyryl fentanyl carboxy metabolite, Butyryl Norfentanyl, Carfentanyl, CUMYL-4CN-BINACA, CUMYL-BICA, CUMYL-PEGACLONE (SGT-151), CUMYL-PICA, CUMYL-THPINACA, Cyclopropylfentanyl, Despropionyl *para*-Fluorofentanyl, Ethylphenidate, Fentanyl, Furanyl norfentanyl, EG-018, EG-2201, Ethcathinone, Ethylone, FDU-PB-22, FUB-JWH-018, FUB-NPB-22, FUB-PB-22, JWH-007, JWH-011, JWH-015, JWH-016, JWH-018, JWH-019, JWH-020, JWH-022, JWH-030, JWH-031, JWH-073, JWH-080, JWH-081, JWH-098, JWH-122, JWH-122 N-(4-pentenyl) analog, JWH-145, JWH-147, JWH-182, JWH-200, JWH-203, JWH-210, JWH-213, JWH-249, JWH-250, JWH-251, JWH-302, JWH-307, JWH-309, JWH-370, JWH-387, JWH-398, JWH-412, JWH-424, M-144, MDMB-4en-PINACA, MDMB-CHMCZCA, MDMB-CHMICA, MDMB-CHMINACA, MDMB-FUBICA, MDMB-FUBINACA, MDMB-PICA, MDMB-PINACA, MDPV, Mephedrone (4-Methyl MCAT, 4-MMC), MEPIRAPIM, Methcatinone (MCAT), Methedrone (4-Methoxy MCAT), Methoxyacetyl norfentanyl, Methylone, MMB-022 (MMB-4en-PICA), MMB-2201(5F-AMB-PICA), MN-25, NE-CHMIMO, N,N-Dimethylcathinone, N,N-Dimethyltryptamine (DMT), Norfentanyl, N-Phenyl-SDB-006, Pentylone, Phenylfentanyl, Phenylacetyl fentanyl, RCS-4, RCS-8, Ritalinic acid, SDB-005, THJ-2201, WIN 48,098 (Pravadoline), WIN 55,212-2, XLR-11, XLR-12.

Analyses were performed by ultra-performance liquid chromatography tandem mass spectrometry (UPLC-MS/MS) on blood samples. The method was validated according to the SWGTOX guidelines [1].

**SUPPLEMENTARY MATERIAL (1) REFERENCES**

[1] Scientific Working Group for Forensic Toxicology (SWGTOX) standard practices for method validation in forensic toxicology. Journal of Analytical Toxicology 37(7):452-474
