# Supplementary material for: Importance of dashboard camera (Dash Cam) analysis in fatal vehicle–pedestrian crash reconstruction
Source: Forensic Sci Med Pathol. 2021 May 19;17(3):379–87. doi: 10.1007/s12024-021-00382-0 (PMC8413177; doi:10.1007/s12024-021-00382-0)
Supplement: Supplementary file 2 — Supplementary file2 (DOCX 56 KB) [file 12024_2021_382_MOESM2_ESM.docx]

**Supplementary material (2) to:**

**Importance of dashboard camera analysis in fatal vehicle-pedestrian crash reconstruction**

In June 2020 was performed a research regarding laws that regulate the use of dashcams worldwide. The search has been performed on Internet search engines (Google) using the terms “dashcam windscreen legislation” followed by the name of the individual state of interest. The research has been performed in several languages: for Europe the keywords have been translated in English, Italian, French, Spanish, German and Portuguese; for North America in English; for Central and South America in Spanish and Portuguese; for Asia, Africa and Oceania in English. For each country, the first 10 sources that appeared on the search engine were included.

**1. Europe**

The General Regulation on Data Protection (GDPR) no. 2016/679 is a European Union regulation on the processing of personal data and privacy, adopted on 27 April 2016 [1]. With this regulation, the European Commission aims to strengthen the protection of personal data of EU citizens. Later, in July 2019, were published the Guidelines no. 3/2019 of the European Data Protection Board (EDPB) [2]. These guidelines deal specifically with the subject of dashcams, stipulating that recordings of traffic or public land should not be continuous, and that any people filmed in the videos should always be informed. Inspired by the legislation of the European Union, each European state, which is to be analyzed, has stipulated a specific regulation of surveillance and privacy [3, 4].

**1.2 Austria**

The Austrian Federal Law on Personal Data Protection has stipulated that only the police and other public authorities may use technological devices for the processing of videos for administrative procedural purposes. Therefore, this decision of the Austrian Personal Data Protection prohibited the private use of dash cams, although video recordings after a possible traffic accident are allowed. Offenders are required to pay penalties of between euros 10,000 and 25,000 [5, 6].

**1.3 Belgium**

In 2014 the Belgian law on privacy protection, in relation to the processing of personal data, has declared that the use of dash cams is permitted, provided that the recorded images are used for private use. It is permitted to use these images as evidence in court during a legal process.

**1.4 Cipro**

In September 2019, the Cyprus Privacy Authority of Nicosia reiterated the absence of a national legal framework governing the use of dash cams and stated that it is the role of the courts to decide on a case-by-case basis on the legality of evidence obtained through dash cams. Furthermore, the Cyprus Privacy Authority has recommended that citizens should always seek the consent of other parties involved [8].

**1.5 France**

According to Articles 226-1 et seq. of the Penal Code, it results that dashcams do not violate privacy. The dashcams must not obstruct the driver's view, the footage is for private use only and cannot be uploaded on the internet, but should be sent directly to the police in the event of a traffic accident [9].

**1.6 Germany**

The Federal Data Protection Act of Germany does not expressly regulate video surveillance and cameras [10]. The law establishes the general principles regarding the legal basis for data processing and the rights of the data subject. The Ansbach Administrative Court has decided that the dashcams are incompatible with German legal acts on data protection; as a result of this decision, the Bavarian Personal Data Protection Authority has decided that the use of the dashcams may result in fines of up to 300,000 euros [7, 11].

**1.7 Ireland**

The Irish Data Protection Commission (DPC) has drawn up a series of guidelines for the use of dashcams. In the car there must be a clearly visible sign or sticker to indicate the presence of a recording device and the footage should only be used for personal use. In the event of a traffic accident, it is necessary to inform the other party about the recording of the footage [12].

**1.8 Italy**

According to Article 2712 of the Civil Code and to Data Protection Supervisor's video surveillance measure of the Italian Republic, the reproduction of video recordings is allowed only with the consent of the counterparts. So, dashcams are lawful, although they must not obstruct the driver's view. Furthermore, videos acquired through dashcams may serve as evidence in court if they are not contested by the other parties [13, 14].

**1.9 Lithuania**

In 2014, the Lithuanian State Data Protection Inspectorate, in agreement with the Director of the Lithuanian State Data Protection Inspectorate, stated that dashcams are allowed in Lithuania, but the publication of data recorded with dashcams can be considered a violation of personal rights, at least if faces and license plates are not recognizable [7].

**1.10 Luxembourg**

In 2013, the National Privacy Protection Commission stated that the implementation of the GDPR no. 2016/679 is incompatible in reality with the use of dashcams, since it would be necessary that the video recordings duration is only a few minutes, that the purposes of the recording are explicit and legitimate and that there is the authorization of all those involved before recording [15]. Although there are no specific legal acts in Luxembourg prohibiting the use of video cameras, the position of the National Commission is sufficiently rigorous and clearly demonstrates that the use of video cameras in this country is prohibited. In fact, recording with a dashcam can result in a fine or, in serious cases, imprisonment [7, 16]^.^

**1.11 Portugal**

Article 19 of Law 58/2019, drafted by the Lisbon National Data Protection Commission, states that the use of dashcams in public places is illegal. The privacy authority of Lusitania also states that offenders will have to pay a fine of between 500-1000 euros [17, 18].

**1.12 Spain**

In Spain there is no rule prohibiting the use of dashcams, unless they obstruct the driver's field of vision, as stipulated in Article 13 of the Law on the Circulation of Motor Vehicles [19]. The Organic Law on Data Protection also states that it is permitted to videotape people in public places, provided that they have given their consent to the use of the images in order to respect their privacy; while uploading images to the internet is prohibited, as the images are for personal use only [20, 21].

**1.13 Switzerland**

The supervisory authority for data protection in Switzerland is the Federal Data Protection and Transparency Commissioner, who has stated that dash cams are legal and that the faces of persons or vehicle registration numbers must be rendered unrecognisable in video recordings [22]. In addition, dash cams may never be used for entertainment, because it must have a legal purpose to record as evidence in case of serious crimes or traffic accidents [23].

**1.14 Russia**

In Russia there has been a large production and use of dashcams as a solution to all types of disputes related to traffic accidents. Finally, in 2009, the Russian Ministry of Interior legalized the use of dashcams as long as they do not obstruct the driver's view [7, 24].

**1.15 United Kingdom**

The UK Data Protection Act regulates how state organisations, businesses or institutions use personal information [25]. The use of dashcams is permitted as long as they are not a distraction for the driver and do not obscure his or her view of the road. For privacy reasons, faces and license plates must be made blurred and unrecognisable. In addition, taxi companies must indicate the presence of dashcams on board the vehicle [7].

**1.16 Other European countries** [26]

- Albania, Bulgaria, Croatia, Czech Republic, Denmark, Greece, Iceland, Norway: the use of dash cams is permitted, but must not in any way impair the driver's vision or ability to drive safely;
- Armenia, Estonia, Malta, Macedonia, Moldova, Montenegro, Netherlands, Poland, Romania, Serbia, Slovenia, Sweden, Turkey, Ukraine: the use of dash cams is permitted but must not impair the driver's vision or ability to drive safely in any way. The publication of movies is allowed if faces and license plates are blurred;
- Andorra, Hungary: The use of dash cams must not impair the driver's vision in any way and is only permitted for private use;
- Slovakia: the use of dash cams must not impair the driver's vision in any way and is only permitted as legal evidence in the event of an accident.

**2. North America**

**2.1** [**United States**](https://en.wikipedia.org/wiki/United_States)

In the United States, video recording of public events is protected at the federal level by First Amendment law, after which individual states stipulate specific laws relating to image and sound recording and the implications for privacy [27]. Regarding the use of dashcams in United States, the states can be divided into three categories:

- Alabama, Alaska, Arizona, Arkansan, California, Connecticut, Florida , Georgia, Hawaii, Idaho, Illinois, Indiana, Iowa, Kansans, Kentucy, Louisiana, Maine, Maryland, Montana, Nevada, New Jersey, North Dakota, Ohio, Oklahoma, Pennsylvania, Rhode Island, South Carolina, South Dakota, Oregon, Tennessee, Texas, West Virginia, Wyoming, Utah: no person shall drive any motor vehicle with any nontransparent material upon the front windshield which materially obstructs, obscures, or impairs the driver’s clear view of the highway [28-61];
- Colorado, Delaware , Massachusetts, Michigan, Minnesota, Mississippi, Nebraska, New Hampshire, New Jersey, New York, New Mexico, Vermont , Virginia, Washington, Wisconsin: no person shall drive any motor vehicle with any non-transparent material upon the front windshield, than a certificate required to be so displayed by regulations of the commissioner [62-76];
- Missouri, North Carolina: no reference is included in the state regulation regarding the use of nontransparent material upon the front windshield [77, 78].

In addition, the use of electronic recording equipment is governed by federal and state laws, under which in thirty-eight states the recording of conversations without the consent of the persons concerned is permitted, while twelve states (California, Connecticut, Florida, Illinois, Maryland, Massachusetts, Michigan, Montana, Nevada, New Hampshire, Pennsylvania and Washington) require the consent of all parties to a conversation. Federal law always makes it illegal to disclose the content of a call without the consent of the parties [79].

**2.2 Canada**

In Canada, the regulation of video recordings of public events is governed by the laws of individual states. Regarding the use of dashcams in Canada, the states can be divided into two main categories:

- Alberta, Manitoba, Newfoundland and Labrador, Northwest Territories, Nunavut, Saskatchewan, Yukon: dashcam mounting is legal and regulated by specific state laws;
- British Columbia, New Brunswick, Nova Scotia, Ontario, Prince Edward Island, Québec: the mounting of dashcams is prohibited because, according to specific state legislation, the use of non-transparent material on the dashcam is prohibited [80].

**3. South America**

**3.1 Brazil**

The regulation of personal data processing in Brazil is governed by Law 4060/12 and Law 13.709/18. The use of dashcams is therefore lawful, even if the processing of sensitive data can only take place with the consent of the person concerned [81].

**3.2 Argentina**

The Ministry of Justice and Human Rights of the Presidency of the Argentine Nation has issued two regulations on the processing of personal data: disposition 238/2012 of the Ministry of Security of the Presidency of the Nation and disposition 10/2015 of the National Directorate for the Protection of Personal Data. The use of dashcams is lawful, as long as there is consent and respect for the privacy of the people filmed [82].

**4. Africa, Asia and Oceania**

- South Africa: dashcams may be used as long as the recording also includes audio of conversations with other people [83];
- Saudi Arabia, Kazakhstan, India, China, Japan: there are no specific laws on dashcams, which can therefore be used for personal use [84-88];
- Australia: there is no specific reference to dashcams in the Federal Government's Privacy Act of 1988, but they would fall into the category of privately operated surveillance cameras. Dashcams are therefore allowed in all states of Australia, provided that the recording does not violate personal privacy [89-90].

**SUPPLEMENTARY MATERIAL (2) REFERENCES**

1. Regulation 2016/679 of the European Parliament and of the Council of 27 April 2016. 2016. <https://eur-lex.europa.eu/eli/reg/2016/679/oj>. Accessed 20 Jun 2020.

2. Guidelines 3/2019 on processing of personal data through video devices. 2019. <https://edpb.europa.eu/sites/edpb/files/consultation/edpb_guidelines_201903_videosurveillance.pdf>. Accessed 20 Jun 2020.

3. Using your dash cam abroad: what you need to know about driving in Europe. 2018. <https://www.which.co.uk/news/2018/08/using-your-dash-cam-abroad-what-you-need-to-know-about-driving-in-europe/>. Accessed 20 Jun 2020.

4. Where are Dash Cams legal across Europe?. https://www.nextbase.com/en-au/where-are-dash-cams-legal-across-europe/Accessed 19 Jun 2020.

5. Beschwerde an die Datenschutzbehörde (DSB). <https://www.dsb.gv.at/fragen-und-antworten#Dashcams_Autokameras>. Accessed 20 Jun 2020.

6. Private Videoüberwachung: Datenschützer warnt vor Selbstjustiz. 2013. <https://helpv2.orf.at/stories/1717004/index.html>. Accessed 18 Jun 2020.

7. Štitilis D, Laurinaitis M. Legal regulation of the use of dashboard cameras: Aspects of privacy protection. Computer Law & Security Review. 2016; https://doi.org/10.1016/j.clsr.2016.01.012

8. Ανακοίνωση Επιτρόπου Προστασίας Δεδομένων Προσωπικού Χαρακτήρα σχετικά με τη χρήση dash cams (καμερών-ταμπλό). 2019. <http://www.dataprotection.gov.cy/dataprotection/dataprotection.nsf/All/943410C63CF6430CC2258470003F0A71>. Accessed 18 Jun 2020.

9. Code pénal, Livre II, Titre II, Chapitre VI, Section 1, Article 226-1. 2002. <https://www.legifrance.gouv.fr/affichCodeArticle.do?idArticle=LEGIARTI000006417929&cidTexte=LEGITEXT000006070719&dateTexte=20020101>. Accessed 20 Jun 2020.

10. Federal Data Protection Act (Bundesdatenschutzgesetz, BDSG). 1994. https://www.iuscomp.org/gla/statutes/BDSG.htm. Accessed 20 Jun 2020.

11. Urteil des VI. Zivilsenats vom 15.5.2018 - VI ZR 233/17. 2018. <http://juris.bundesgerichtshof.de/cgi-bin/rechtsprechung/document.py?Gericht=bgh&Art=en&nr=85141&pos=0&anz=1>. Accessed 20 Jun 2020.

12. An Coimisiùn um Chosaint Sonraì, Data Protection Commision. 2011. <https://www.dataprotection.ie/en>. Accessed 20 Jun 2020.

13. Codice civile, Art. 2712. 1942. <https://www.brocardi.it/codice-civile/libro-sesto/titolo-ii/capo-ii/sezione-iv/art2712.html>. Accessed 17 Jun 2020.

14. Garante per la protezione dei dati personali, Repubblica Italiana. 2010. <https://www.garanteprivacy.it/home/docweb/-/docweb-display/docweb/1712680>. Accessed 19 Jun 2020.

15. National Commision for Data Protection. Gran-Duchy of Luxembourg. https://cnpd.public.lu/en.html. Accessed 20 Jun 2020.

16. Les caméras de surveillance installées dans les voitures sont-elles licites?. 2013. <https://gouvernement.lu/fr/actualites/toutes_actualites/communiques/2013/06-juin/21-camera-voitures.html>. Accessed 19 Jun 2020.

17. Lei n.58/2019, de 08 de Agosto. Lei da proteção de dados pessoais. 2019. <http://www.pgdlisboa.pt/leis/lei_mostra_articulado.php?nid=3118&tabela=leis&ficha=1&pagina=1&so_miolo=>. Accessed 20 Jun 2020.

18. Uso de câmaras de vídeo em carros está proibido por lei, confirma CNPD. 2019. <https://visao.sapo.pt/exameinformatica/noticias-ei/mercados/2019-11-11-Uso-de-camaras-de-video-em-carros-esta-proibido-por-lei-confirma-CNPD/>. Accessed 18 Jun 2020.

19. Infracciones según art. 17 y 18 del reglamento general de circulación. 2017. <http://legaccidentes.com/infracciones-segun-art-17-y-18-del-reglamento-general-de-circulacion/>. Accessed 20 Jun 2020.

20. Ley Orgánica 3/2018, de 5 de diciembre, de Protección de Datos Personales y garantía de los derechos digitales. 2018. <https://www.boe.es/buscar/pdf/2018/BOE-A-2018-16673-consolidado.pdf>. Accessed 20 Jun 2020.

21. ¿Sabes qué es una dashcam o si es legal en España?. 2017. <https://www.legalitas.com/actualidad/Sabes-que-es-una-dashcam-o-si-es-legal-en-Espana>. Accessed 17 Jun 2020.

22. Lea, Protection des données. https://www.lea-label.ch/it/protezione-dei-dati/. Accessed 20 Jun 2020.

23. Explications relatives aux caméras de bord. 2019. <https://www.edoeb.admin.ch/edoeb/it/home/protezione-dei-dati/technologien/videoueberwachung/spiegazioni-sulla-videosorveglianza-nei-veicoli--dashcam-.html>. Accessed 17 Jun 2020.

24. [Приказ МВД № 185 Об утверждении административного регламента МВД"](http://www.stopca.ru/zakony/prikaz_mvd__185_ob_utverzhdenii_administrativnogo_reglamenta_mvd/). 2009. <http://www.stopca.ru/zakony/prikaz_mvd__185_ob_utverzhdenii_administrativnogo_reglamenta_mvd/>. Accessed 20 Jun 2020.

25. Data Protection Act 1998, UK Public General Acts. 1998. <http://www.legislation.gov.uk/ukpga/1998/29/contents>. Accessed 19 Jun 2020.

26. Where are Dash Cams legal across Europe?. https://www.nextbase.com/en-au/where-are-dash-cams-legal-across-europe/. Accessed 20 Jun 2020.

27. LII U.S. Constitution First Amendment. <https://www.law.cornell.edu/constitution/first_amendment>. Accessed 20 Jun 2020.

##### 28. 2006 Alabama Code - Section 32-5-215 — Windshields must be unobstructed; windshield wipers; tinting. 2006. <https://law.justia.com/codes/alabama/2006/22786/32-5-215.html>. Accessed 20 Jun 2020.

29. 13 Alaska Admin Code § 04.225. 2020. <https://casetext.com/regulation/alaska-administrative-code/title-13-public-safety/part-1-division-of-alaska-state-troopers/chapter-04-motor-vehicle-and-driving-offenses-vehicle-equipment-and-inspection/article-4-brakes-and-other-equipment/section-13-aac-04225-windshields-and-wipers>. Accessed 19 Jun 2020.

30. 28-959.01 Materials on windows or windshield; exceptions; requirements; violation; definitions. <https://www.azleg.gov/ars/28/00959-01.htm>. Accessed 19 Jun 2020.

31. 2016 Arkansas Code Title 27 – Transportation Subtitle 3 - Motor Vehicles and Their Equipment Chapter 37 - Equipment Regulations Subchapter 3 - -- Glass and Mirrors § 27-37-302. Windshields, etc., to be unobstructed. 2016. <https://law.justia.com/codes/arkansas/2016/title-27/subtitle-3/chapter-37/subchapter-3/section-27-37-302/>. Accessed 18 Jun 2020.

32. 2009 California Vehicle Code - Section 26700-26712: Chapter 4. Windshields And Mirrors. 2009. <https://law.justia.com/codes/california/2009/veh/26700-26712.html>. Accessed 19 Jun 2020.

33. Connecticut General Statutes 14-99f - Windshield. Obstruction of view. 2020. <https://www.lawserver.com/law/state/connecticut/ct-laws/connecticut_statutes_14-99f>. Accessed 20 Jun 2020.

34. 2014 Florida Statutes TITLE XXIII - MOTOR VEHICLES Chapter 316 - STATE UNIFORM TRAFFIC CONTROL 316.2004 - Obstruction to driver’s view or driving mechanism. 2014. <https://law.justia.com/codes/florida/2014/title-xxiii/chapter-316/section-316.2004/>. Accessed 19 Jun 2020.

35. 2016 Georgia Code Title 40 - Motor Vehicles and Traffic Chapter 8 - Equipment and Inspection of Motor Vehicles. 2016. <https://law.justia.com/codes/georgia/2016/title-40/chapter-8/article-1/part-4/section-40-8-73/>. Accessed 17 Jun 2020.

36. §291-21.5 Regulation of motor vehicle sun screening devices; penalty. <https://www.capitol.hawaii.gov/hrscurrent/Vol05_Ch0261-0319/HRS0291/HRS_0291-0021_0005.htm>. Accessed 19 Jun 2020.

37. Idaho Statutes. <https://legislature.idaho.gov/statutesrules/idstat/Title49/T49CH9/SECT49-943/>. Accessed 19 Jun 2020.

38. 2010 Illinois Code CHAPTER 625 VEHICLES 625 ILCS 5/ Illinois Vehicle Code. Article V - Glass, Windshields And Mirrors. 2010. <https://law.justia.com/codes/illinois/2010/chapter625/062500050HCh_12_Art_V.html>. Accessed 20 Jun 2020.

39. 2012 Indiana Code TITLE 9. MOTOR VEHICLES ARTICLE 19. MOTOR VEHICLE EQUIPMENT CHAPTER 19. WINDOWS AND WINDSHIELD WIPERS. 2012. <https://law.justia.com/codes/indiana/2012/title9/article19/chapter19/>. Accessed 19 Jun 2020.

40. 1 MOTOR VEHICLES AND LAW OF THE ROAD, §321.438. <https://www.legis.iowa.gov/docs/code/2018/321.438.pdf>. Accessed 18 Jun 2020.

41. 2012 Statute. 2012. <http://kslegislature.org/li_2012/b2011_12/statute/008_000_0000_chapter/008_017_0000_article/008_017_0041_section/008_017_0041_k/>. Accessed 17 Jun 2020.

42. KRS 189.110 Unobstructed windshields — Display of American flag — Windshield wipers. 2016. <https://lawreader.com/?p=15985>. Accessed 19 Jun 2020.

43. RS 32:361.1. <http://legis.la.gov/Legis/law.aspx?d=88294>. Accessed 20 Jun 2020.

44. Title 29-A: MOTOR VEHICLES AND TRAFFIC Chapter 19: OPERATION. <https://mainelegislature.org/legis/statutes/29-A/title29-Asec2082.html>. Accessed 18 Jun 2020.

45. 2013 Maryland Code TRANSPORTATION § 22-404 - Windshields must be unobstructed and equipped with wipers. 2013. <https://law.justia.com/codes/maryland/2013/article-gtr/section-22-404/>. Accessed 19 Jun 2020.

46. Montana Code. <https://leg.mt.gov/bills/mca/61/9/61-9-405.htm>. Accessed 17 Jun 2020.

47. CHAPTER 484D - EQUIPMENT, INSPECTIONS AND SIZE, WEIGHT AND LOAD OF VEHICLES. <https://www.leg.state.nv.us/NRS/NRS-484D.html#NRS484DSec435>. Accessed 19 Jun 2020.

48. 2013 New Jersey Revised Statutes Title 39 - MOTOR VEHICLES AND TRAFFIC REGULATION Section 39:3-74 - Windshields must be unobstructed and equipped with cleaners. 2013. <https://law.justia.com/codes/new-jersey/2013/title-39/section-39-3-74>. Accessed 20 Jun 2020.

49. CHAPTER 39-21 EQUIPMENT OF VEHICLES. <https://www.legis.nd.gov/cencode/t39c21.pdf>. Accessed 19 Jun 2020.

50. Ohio Code. <http://codes.ohio.gov/orc/4511>. Accessed 18 Jun 2020.

51. 2014 Oklahoma Statutes Title 47. Motor Vehicles §47-12-404. Windshields and windows - Obstruction, obscuring, or impairing of driver's view - Electric windshield wiper mechanism. <https://law.justia.com/codes/oklahoma/2014/title-47/section-47-12-404/>. Accessed 19 Jun 2020.

52. 2010 Pennsylvania Code Title 75 – VEHICLES Chapter 45 - Other Required Equipment 4524 - Windshield obstructions and wipers. 2010. <https://law.justia.com/codes/pennsylvania/2010/title-75/chapter-45/4524/>. Accessed 19 Jun 2020.

# 53. 2012 Rhode Island General Laws Title 31 - Motor and Other vehicles Chapter 31-23. 2012. <https://law.justia.com/codes/rhode-island/2012/title-31/chapter-31-23/chapter-31-23-16/>. Accessed 17 Jun 2020.

54. 2012 South Carolina Code of Laws Title 56 - Motor Vehicles Chapter 5 - UNIFORM ACT REGULATING TRAFFIC ON HIGHWAYS Section 56-5-5000 - Windows shall be unobstructed; windshield wipers.. 2012. <https://law.justia.com/codes/south-carolina/2012/title-56/chapter-5/section-56-5-5000/>. Accessed 19 Jun 2020.

55. South Dakota Code (§ 32-15). <https://sdlegislature.gov/Statutes/Codified_Laws/DisplayStatute.aspx?Type=Statute&Statute=32-15>. Accessed 20 Jun 2020.

56. ORS 815.220 Obstruction of vehicle windows. 2020. <https://www.oregonlaws.org/ors/815.220>. Accessed 19 Jun 2020.

57. 2016 Tennessee Code Title 55 - Motor and Other Vehicles Chapter 8 - Operation of Vehicles -- Rules of the Road. 2016. <https://law.justia.com/codes/tennessee/2016/title-55/chapter-8/part-1/section-55-8-165/>. Accessed 19 Jun 2020.

58. TRANSPORTATION CODE TITLE 7. VEHICLES AND TRAFFIC. <https://statutes.capitol.texas.gov/Docs/TN/htm/TN.547.htm>. Accessed 18 Jun 2020.

59. 2012 West Virginia Code CHAPTER 17C. TRAFFIC REGULATIONS AND LAWS OF THE ROAD. 2012. <https://law.justia.com/codes/west-virginia/2012/chapter17c/article14/17c-14-4/>. Accessed 17 Jun 2020.

# 60. 2011 Wyoming Statutes TITLE 31 - MOTOR VEHICLES CHAPTER 5 - REGULATION OF TRAFFIC ON HIGHWAYS 31-5-955. Windshields and wipers. 2011. <https://law.justia.com/codes/wyoming/2011/title31/chapter5/section31-5-955/>. Accessed 19 Jun 2020.

61. 2018 Utah CodeTitle 41 - Motor Vehicles Chapter 6a - Traffic Code Part 16 - Vehicle Equipment. 2018. <https://law.justia.com/codes/utah/2018/title-41/chapter-6a/part-16/section-1635/>. Accessed 20 Jun 2020.

62. Colorado Revised Statutes Title 42. Vehicles and Traffic § 42-4-227. Windows unobstructed--certain materials prohibited--windshield wiper requirements. <https://codes.findlaw.com/co/title-42-vehicles-and-traffic/co-rev-st-sect-42-4-227.html>. Accessed 19 Jun 2020.

63. Motor Vehicles Operation and Equipment CHAPTER 43. Equipment and Construction of Vehicles. <https://delcode.delaware.gov/title21/c043/sc01/index.shtml#4309>. Accessed 18 Jun 2020.

64. 2006 Massachusetts Code - Chapter 90 — Section 9D. Windshields and windows obscured by nontransparent materials. 2006. <https://law.justia.com/codes/massachusetts/2006/gl-pt1-toc/90-9d.html>. Accessed 19 Jun 2020.

65. MICHIGAN VEHICLE CODE (EXCERPT) Act 300 of 1949. <http://www.legislature.mi.gov/(S(rck5oys4in13jdtmuo2ab5g4))/mileg.aspx?page=getObject&objectName=mcl-257-709>. Accessed 19 Jun 2020.

66. 2017 Mississippi Code Title 63 - Motor Vehicles and Traffic Regulations Chapter 7 - Equipment and Identification

General Provisions. 2017. <https://law.justia.com/codes/mississippi/2017/title-63/chapter-7/general-provisions/section-63-7-59/>. Accessed 20 Jun 2020.

67. 2020 Minnesota Statutes. 2020. <https://www.revisor.mn.gov/statutes/cite/169.71>. Accessed 17 Jun 2020.

68. 2013 Nebraska Revised Statutes Chapter 60 - MOTOR VEHICLES 60-6,255 - Windshield and windows; nontransparent material prohibited; windshield equipment; requirements. 2013. <https://law.justia.com/codes/nebraska/2013/chapter-60/statute-60-6-255>. Accessed 18 Jun 2020.

69. 2015 New Hampshire Revised Statutes Title XXI - MOTOR VEHICLES Chapter 265 - RULES OF THE ROAD

Section 265:95 - Obstruction to Driver's View or Riding Mechanism. 2015. <https://law.justia.com/codes/new-hampshire/2015/title-xxi/chapter-265/section-265-95/>. Accessed 19 Jun 2020.

70. 2013 New Jersey Revised Statutes Title 39 - MOTOR VEHICLES AND TRAFFIC REGULATION Section 39:3-74 - Windshields must be unobstructed and equipped with cleaners. 2013. <https://law.justia.com/codes/new-jersey/2013/title-39/section-39-3-74>. Accessed 20 Jun 2020.

71. 2013 New York Consolidated Laws VAT - Vehicle & Traffic Title 3 - SAFETY RESPONSIBILITY; FINANCIAL SECURITY; EQUIPMENT; INSPECTION; SIZE AND WEIGHT; AND OTHER PROVISIONS. 2013. <https://law.justia.com/codes/new-york/2013/vat/title-3/article-9/375/>. Accessed 18 Jun 2020.

72. 2011 New Mexico Statutes Chapter 66: Motor Vehicles. 2011. <https://law.justia.com/codes/new-mexico/2011/chapter66/article3/section66-3-846/>. Accessed 19 Jun 2020.

73. 2013 Vermont Statutes Title 23 Motor Vehicles Chapter 13 OPERATION OF VEHICLES § 1125 Obstructing windshields. 2013. <https://law.justia.com/codes/vermont/2013/title-23/chapter-13/section-1125/>. Accessed 19 Jun 2020.

74. § 46.2-1052. (Effective until March 1, 2021) Tinting films, signs, decals, and stickers on windshields, etc.; penalties. 2020. <https://law.lis.virginia.gov/vacode/title46.2/chapter10/section46.2-1052/>. Accessed 17 Jun 2020.

75. RCW 46.37.410 Windshields required, exception-Must be unobstructed and equipped with wipers. https://app.leg.wa.gov/rcw/default.aspx?cite=46.37.410. Accessed 19 Jun 2020.

76. Wisconsin State Legislature. <https://docs.legis.wisconsin.gov/code/admin_code/trans/305/II/32>. Accessed 20 Jun 2020.

77. Missouri Code. <http://revisor.mo.gov/main/OneChapter.aspx?chapter=307>. Accessed 20 Jun 2020.

78. Chapter 20. Motor Vehicles. <https://www4.ncleg.net/enactedlegislation/statutes/html/bychapter/chapter_20.html>. Accessed 19 Jun 2020.
79. Reporter’s Recording Guide. Reporters Committee for freedom of the press. 2012. <https://www.rcfp.org/wp-content/uploads/imported/RECORDING.pdf>. Accessed 20 Jun 2020.

80. Windshield Law And The GPS Receiver. 2012. <http://www.poi-factory.com/node/34521>. Accessed 19 Jun 2020.

81. Regulamentação do tratamento de dados pessoais foi destaque entre aprovações da área de comunicação. Camara dos deputatos. 2019. <https://www.camara.leg.br/noticias/550227-regulamentacao-do-tratamento-de-dados-pessoais-foi-destaque-entre-aprovacoes-da-area-de-comunicacao/>. Accessed 20 Jun 2020.

82. SID 2015, XV Simposio Argentino de Informática y Derecho.  Estado de la normativa sobre video vigilancia en Argentina y su relación con la protección de datos personales. 2015. <http://sedici.unlp.edu.ar/bitstream/handle/10915/55549/Documento_completo.pdf-PDFA.pdf?sequence=1&isAllowed=y>. Accessed 17 Jun 2020.

83. Are dashcams actually legal in South Africa? 2017. <https://businesstech.co.za/news/motoring/154387/are-dashcams-actually-legal-in-south-africa/>. Accessed 20 Jun 2020.

84. Prove your case with a ‘Dashcam’. <https://www.arabnews.com/motoring/news/727976>. 2015. Accessed 18 Jun 2020.

85. Guide to Driving In Kazakhstan. <https://www.rhinocarhire.com/Drive-Smart-Blog/Drive-Smart-Kazakhstan.aspx#/searchcars>. Accessed 20 Jun 2020.

86. Car Dash Cam: why Should You Invest In It. <https://gomechanic.in/blog/car-dash-cam-india/>. 2019. Accessed 20 Jun 2020.

87. Cybersecurity Law of the People’s Republic of China. 2017. <https://iapp.org/resources/article/cybersecurity-law-of-the-peoples-republic-of-china-english-translation/>. Accessed 17 Jun 2020.

88. Japan The Act on the Protection of Personal Information. 2003. <http://www.cas.go.jp/jp/seisaku/hourei/data/APPI.pdf>. Accessed 20 Jun 2020.

89. Privacy Act 1988, Federal Register of Legislation. 1988. <https://www.legislation.gov.au/Series/C2004A03712>. Accessed 19 Jun 2020.

90. Dascams Australia FAQ. <https://dashcamsaustralia.com.au/faq/>. Accessed 20 Jun 2020.
